# Development of Buccal Adhesive Tablet with Prolonged Antifungal activity: Optimization and *ex vivo* Deposition Studies

**DOI:** 10.4103/0250-474X.56032

**Published:** 2009

**Authors:** A. Madgulkar, S. Kadam, V. Pokharkar

**Affiliations:** Bharati Vidyapeeth University, Poona College of Pharmacy, Paud Road, Erandwane, Pune-411 038, India

**Keywords:** Bioadhesion, buccal release, prolonged release miconazole tablets

## Abstract

The purpose of the present work was to prepare buccal adhesive tablets of miconazole nitrate. The simplex centroid experimental design was used to arrive at optimum ratio of carbopol 934P, hydroxypropylmethylcellulose K4M and polyvinylpyrollidone, which will provide desired drug release and mucoadhesion. Swelling index, mucoadhesive strength and *in vitro* drug release of the prepared tablet was determined. The drug release and bioadhesion was dependent on type and relative amounts of the polymers. The optimized combination was subjected to *in vitro* antifungal activity, transmucosal permeation, drug deposition in mucosa, residence time and bioadhesion studies. IR spectroscopy was used to investigate any interaction between drug and excipients. Dissolution of miconazole from tablets was sustained for 6 h. based on the results obtained, it can be concluded that the prepared slow release buccoadhesive tablets of miconazole would markedly prolong the duration of antifungal activity. Comparison of *in vitro* antifungal activity of tablet with marketed gel showed that drug concentrations above the minimum inhibitory concentration were achieved immediately from both formulations but release from tablet was sustained up to 6 h, while the gel showed initially fast drug release, which did not sustain later. Drug permeation across buccal mucosa was minimum from the tablet as well as marketed gel; the deposition of drug in mucosa was higher in case of tablet. *In vitro* residence time and bioadhesive strength of tablet was higher than gel. Thus the buccoadhesive tablet of miconazole nitrate may offer better control of antifungal activity as compared to the gel formulation.

The term bioadhesion is used to describe the attachment of synthetic or natural polymers to biological surface. If the adhesion surface is mucous membrane coated with a thin layer of mucus, the term mucoadhesion is employed[1,2]. Adhesion to specific sites such as oral and nasal cavities increase bioavailability by virtue of optimum contact with adhesion surface which increases absorption of drug and prolongs gastric residence. Recent years have seen an increasing interest in the development of novel mucoadhesive buccal dosage form used both for systemic delivery of drug as well as for local treatment of buccal cavity. The bioadhesive potential of different materials is an important parameter and to determine the same several techniques are reported[3]. Commonly they are based on tensile testing for evaluation of the strength of mucoadhesive interactions as well as specific contact time[4].

Miconazole nitrate is a broad-spectrum antifungal agent that has been extensively applied for the management of dermal[5], buccal[6] and vaginal candidiasis[7]. Gels containing miconazole are currently used. However since the drug does not persist in the oral cavity, gels have to be applied several times a day[8].

The objective of the present study was to prepare and evaluate buccoadhesive miconazole tablets using simplex centroid experimental design. Mixture designs are statistical designs useful when characteristics of formulations are not dependent on quantities of materials used but on their proportions[4,5]. Here the sum total of the proportions of all the excipients is unity and none of the fractions can be negative. Therefore, the levels of different components can be varied with the restriction that the sum total should not exceed one. The formulation was compared with marketed gel preparation on the basis of parameters like *in vitro* dissolution, *in vitro* antifungal activity, permeation and drug deposition in buccal mucosa.

## MATERIALS AND METHODS

Miconazole nitrate was provided by Sankalp Health Care, Karad, Maharashtra, India. The polymers HPMC K4M, Carbopol 934P and polyvinylpyrollidone (PVP K30) were obtained as gift samples from Ranbaxy Research Centre, Jejury, India. The marketed miconazole gel preparation containing 2% miconazole nitrate was purchased from a local pharmacy.

### Preparation of buccoadhesive miconazole tablets:

Tablets were prepared by direct compression using a flat face 8 mm punch. Each tablet contained 10 mg of miconazole nitrate, mannitol, talc and various proportions of bioadhesive polymers carbopol, HPMC K4M and PVP (Table 1). The tablet weight was adjusted for approximately 100 mg.

**TABLE 1:** DOE PARAMETER SETTINGS INCLUDING INGREDIENTS, AND PROPORTION (%) IN THE MIXTURE OF EXCIPIENTS

Contents	A	B	C	AB	BC	AC	ABC
Drug	10	10	10	10	10	10	10
Carbopol	50	-	-	25	25	-	16.6
HPMC	-	50	-	25	-	25	16.6
PVP	-	-	50	-	25	25	16.6
Mannitol	37	37	37	37	37	37	37
Talc	3	3	3	3	3	3	3

Coded level of variables are either low = 0 or 1= 50 mg of total polymer concentration of A, which is the fraction of carbopol or B the fraction of HPMC or C the fraction of PVP or a combination of these as indicated in the table for formulations

### *In vitro* bioadhesive strength measurement:

The *in vitro* bioadhesion studies were conducted using a modification of a biodhesion test assembly described by Gupta *et al*[9]. The tablet was lowered onto the model substrate under constant weight of 5 g for a total contact period of 5 min. Bioadhesive strength was assessed in terms of weight (g) required to detach the tablet from the membrane. All measurements were carried out at 37±0.5°. Porcine buccal mucosa was used as the model membrane. The mucosa was kept frozen in phosphate buffer (PB) pH 6.8 and thawed to room temperature before use. The mucosal membrane was excised by removing the underlying connective and adipose tissue and was equilibrated at 37±1° for 30 min in phosphate buffer (pH 6.8) before the bioadhesion evaluation study.

### *In vitro* drug release study:

Miconazole nitrate release from the prepared buccoadhesive tablets was determined using standard USP type II dissolution test apparatus containing 900 ml of phosphate buffer pH 6.8 maintained at 37±0.5°. Samples of 5 ml were withdrawn at predetermined time intervals over 6 h and replaced with equal volumes of the dissolution medium equilibrated at the same temperature; drug concentration of withdrawn samples was analyzed after filtration (0.45 μ millipore) by UV spectroscopy at 220 nm.

### *Ex vivo* transmucosal permeation studies:

Permeation studies were carried out using a vertical diffusion cell. Porcine buccal mucosa was surgically excised from freshly sacrificed animal, the underlying muscle and connective tissue was cleaned off. Buccal mucosa was mounted on one half-cell of the permeation cell, with its serosal surface facing the receptor solution compartment. Buccal mucosa was secured on the inner side of a mucoadhesive tablet-holding device, and the tablet was then adhered directly onto the buccal mucosa surface. Similarly gel, containing 10 mg of miconazole was applied on another piece of mucosa. The device was then clamped between the two half cells, such that the other side of the tablet was in close contact with the buccal mucosa. The receptor compartment contained 20 ml of phosphate buffer of pH 6.8. The exposed surface area for buccal membrane permeation was 3.14 cm^2^. Permeation experiments were carried out at 37±0.5° for a period of 6 h. Aliquots of 2 ml were withdrawn and were replaced with fresh phosphate buffer at preset intervals and analyzed by UV spectrophotometer. The membrane was digested with methanol for 6 h, sonicated and the drug content was analyzed in filtered portions of digest.

### *In vitro* residence time:

The *in vitro* residence time for tablet and gel was determined using a locally modified USP disintegration apparatus, based on the apparatus reported by Nakamura *et al*[10]. The disintegration medium was composed of 800 ml pH 6.75 isotonic phosphate buffer (IPB) maintained at 37°. A segment of porcine buccal mucosa, 3 cm length, was glued to the glass slab. The tablet surface was hydrated using 15 μl pH 6.8 IPB and then the hydrated surface was brought into contact with the mucosal membrane. The glass slab was vertically fixed to the patch was completely immersed in the buffer solution at the lowest point and was out at the highest point. The time necessary for complete erosion or detachment of the patch from the mucosal surface was recorded (mean of triplicate determinations).

### *In vitro* antifungal activity[11,12]:

Concentration of miconazole nitrate in the simulated salivary solution was determined by measuring the diameter (mm) of the growth inhibition of *C. albicans* as follows. Aliquots of 100 μl of each sample were carefully pipetted into uniformly spaced 7 mm diameter well of the sabrose dextrose potato agar plates. The diameter (mm) of the growth inhibition zone surrounding each agar well inoculated with *C. albicans* was measured, and the concentration of miconazole nitrate was determined from the standard calibration curve constructed under identical conditions. The mean of two determination of each saliva sample was determined.

The agar plates were prepared and sterilized by autoclaving (at 15 lb pressure and 121°) for 15 min. After cooling, 80 mg of gentamycin and 500 mg of ampicillin each in 2 ml sterile solution were added to the agar solution and mixed before pouring into sterile petri dishes. The agar plates are then allowed to cool and solidify at room temperature; then they were inoculated (cultured) with *C. albicans* by using sterile swabs and a five-dimension method. In the proposed method, antibiotics were added to eliminate any possible interference with the assay from nonpathogenic bacteria normally found in the saliva.

### IR Spectroscopic studies:

Presence of any interaction between the drug and excipients used was investigated by Fourier-transform infrared (FTIR) spectroscopy (Jasco 460 plus) by diffused cell technique. The spectra were recorded over the wave number 4000 to 400 cm^−1^.

## RESULTS AND DISCUSSION

Three constituents HPMC, carbopol 934P and PVP were mixed according to mixture design, with multiple constraints on the component proportions, using fixed intervals, as stated in Table 1. The total amount of excipients was maintained at 50 mg, and the miconazole content was 10 mg. The total weight of tablet was adjusted to 100 mg with mannitol and talc. The coordinates of the 7 design points were generated and the respective tablet formulations were prepared.

**Fig. 1:**
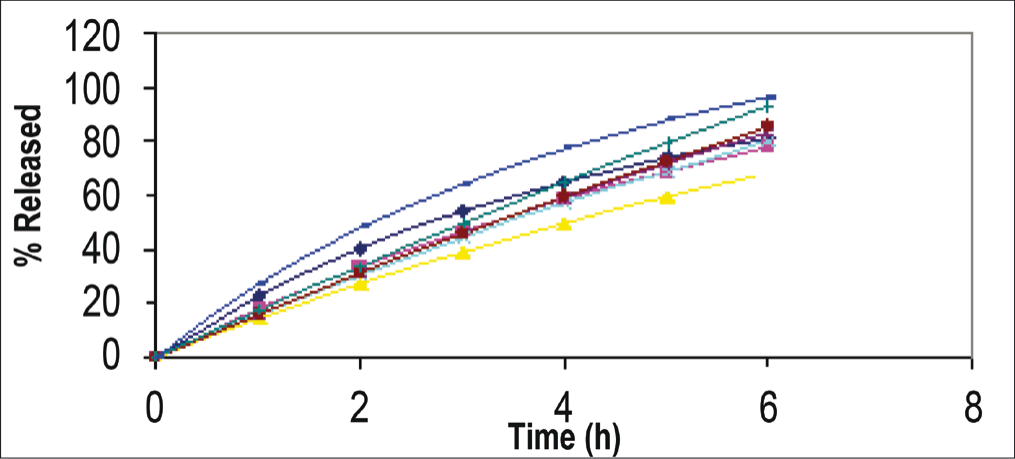
Drug release Profiles of Prepared and optimized formulations. Drug release profiles of prepared and optimized formulations prepared as per simplex centroid design.

**Fig. 2:**
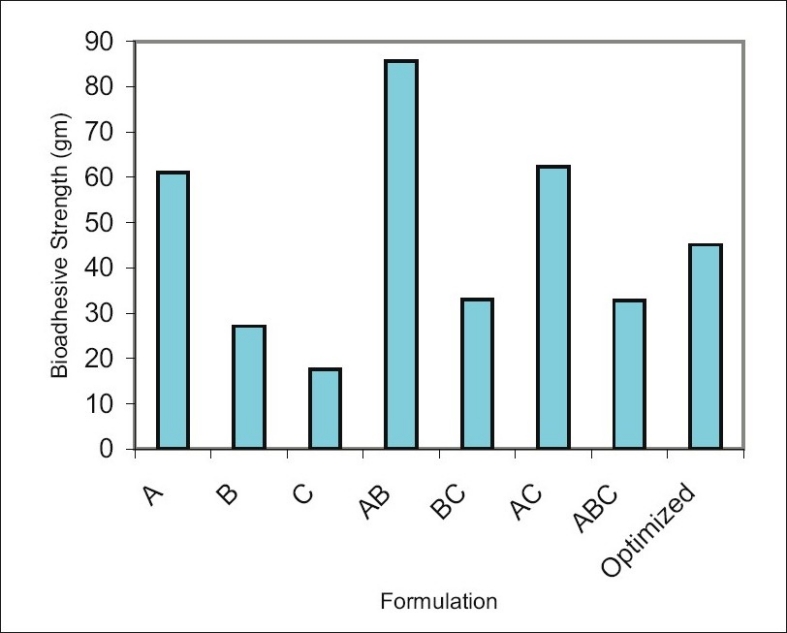
Bioadhesive strength of formulations. Bioadhesive strength of formulations prepared as per simplex centroid design.

**Fig. 3:**
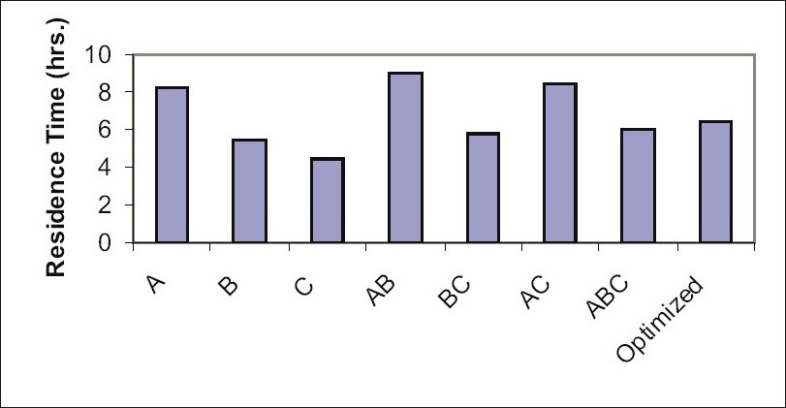
*In vitro* bioadhesion time measurment of formulations. *In vitro* bioadhesion time measurment of formulations prepared in simplex centroid design.

Optimization is applied for finding a factor combination matching an optimal response profile[13]. The design supporting a linear model is useful when the experimental objective is screening, whereas, the design supporting quadratic or special cubic models are relevant for optimization. The mucoadhesive tablets were prepared using optimized formula employing simplex centroid design, which is a useful statistical design for formulation situations where relative proportion of ingredients affects the performance of product, whereas total amount of ingredients remains constant[14]. In the present work a design was constructed for investigating the influence of polymers on the responses (mucoadhesion force and % drug release). With three factors, the lead number of experiments N=7 was constructed and six runs were randomly set. After producing and analyzing the formulations, responses were applied to fit the appropriate model (linear or quadratic). The drug release, bioadhesive strength and *in vitro* bioadhesion time profiles are depicted in figs. 1–3. The model was tested for goodness of fit (*R*^2^) and analysis of variance (ANOVA) was applied to verify the adequacy of the regression model in terms of a lack-of-fit test. It was found that, linear or quadratic models were not significant as presented in Table 2 consequently, special cubic model was used.

**TABLE 2:** ANALYSIS OF VARIANCE OF THE LINEAR AND QUADRATIC MODELS FOR THE RESPONSES

Model	Bioadhesion Force	% Drug Release (6 hours)
Linear	Not Sig	Not Sig
*R*^2^	0.4254	0.2827
*F* value	0.0112	0.2839
Quadratic	Not Sig	Not Sig
*R*^2^	0.5624	0.2859
*F* value	0.3091	0.2595
Special Cubic	Sig	Sig
*R*^2^	0.9984	0.9458
*F* value	0.9368	0.7082

Sig = significant, Not sig = not significant

The following Eqns. 1 and 2 were generated for *in vitro* bioadhesion time and % release respectively, bioadhesion= 61.16*X*_1_ +27.16*X*_2_ +17.61*X*_3_ +186.16*X_1_X_2_* +42.6*X*_2_*X*_3_ +91.96*X*_1_*X*_3_ -1725.2*X*_1_*X*_2_*X*_3_ - (1). This equation shows negative value for combined effect of carbopol, HPMC and PVP on bioadhesion. Special cubic model was also obtained for % drug release and the equation calculated from the regression data was, % Release = 81.23*X*_1_ +78.33*X*_2_ +68.23*X*_3_ +0.8 *X*_1_ *X*_2_ +42.00*X*_1_*X*_3_ +40.80*X*_2_*X*_3_ +223.05*X*_1_*X*_2_*X*_3_ -(2).

Based on the fitted regression models, optimal factor settings were selected by Design-Expert® in order to identify experimental settings in which all desirabilities were met as well as possible. An optimum formulation was generated by the software, which was produced and analyzed for desired response variable. The optimized formula calculated thus contains miconazole 10 mg, carbopol 20 mg, HPMC 20 mg, PVP 10 mg, mannitol 37 mg and talc 3 mg. The predicted and observed results for the optimized tablet formulation shown in Table 3, illustrate that the proposed model has good predictive ability.

**TABLE 3:** PREDICTED AND OBSERVED RESPONSES FROM OPTIMIZED TABLET FORMULATION (N = 3)

Test	Predicted	Observed
Bioadhesion Force	45.15	42.32±2.3654
% Drug Release (6 hours)	91.35 %	96.44 ±3.6781%

The drug release profile shows sustained release over a 6 h period. Thus with the aid of this design it was possible to meet all the specifications, demonstrating that the experimental planning of mixture can supply trustworthy results, reducing the time spent and the number of experimental runs. The swelling studies carried out in phosphate buffer. *Ex vivo* Permeation studies carried out using diffusion cell across buccal mucosa show that 13.01% and 11.87% drug permeated through mucosa from tablet and gel respectively when adhered to buccal mucosa but the amount of drug deposited in buccal mucosa from tablet (40%) was four times higher than that from gel (10%) (Table 4). Dowly *et al.*[15] reported that drop in viability measured from ATP levels did not show a corresponding drop in tissue permeability hence freshly excised porcine buccal mucosa was used for studies. The drug permeation across buccal mucosa was similar from the tablet as well as gel. Thus it can be concluded that the drug does not permeate across the mucosa and is released in the saliva which can provide local action. Four times greater amount of drug was also found to be localized in the mucosa when administered through tablet, this may be explained on the basis of intimate and prolonged contact of tablet as compared to gel. Thus mucoadhesive tablets can provide better action over the gel.

**TABLE 4:** TRANSMUCOSAL PERMEATION STUDY

Time (h)	% Drug Permeate from Tablet	% Drug Permeate from Gel
1	1.93±0.4333	2.48±0.1987
2	4.65±0.5442	3.98±0.5343
3	7.72±0.4306	7.15±0.5453
4	8.14±1.202	9.24±0.6333
5	12.41±1.356	11.56±0.9475
6	13.01±1.468	12.87±0.9857

The *in vitro* residence time (5 h) which was much higher than gel (20 min) may be one of the responsible factors for this. Results of *in vitro* antifungal activity were good for both of the dosage forms but buccoadhesive tablet of miconazole showed sustained action and drug release at the end of 5 h was 25.72 μg/ml while from gel formulation the drug release was initially fast (21.5%) and remained constant onwards (Table 5). *In vitro* antifungal activity determined from zone of inhibition of *C. albicans* showed the drug concentrations above MIC were achieved immediately and was maintained for 5 h while the gel showed initially higher concentrations which declined faster than the tablet. In earlier studies polyethylene glycol gels containing carbopol and PVP have been reported to exhibit pseudoplastic flow with thixotropy, indicating a general loss of consistency with increased shearing stress. The drug release correlates with gel rheology, thus explaining the abrupt increase in drug concentrations from gel formulation. Optimum wetting is important for bioadhesion overhydration results in the formation of wet slippery mucilage without adhesion[16]. Presence of interactions was studied with help of IR. The spectrum for HPMC shows peak corresponding to OH at 3000-3100 cm^−1^, carbopol shows peak corresponding to C=O in viscinity of 1600-1700 cm^−1^. While in the blend both the peaks appear with reduced intensity. Thereby, confirming the absence of interaction between polymers. Investigators reported interaction between polymers in acidic pH but since pH involved in study was 6.8 the interaction may not have taken place[17].

**TABLE 5:** *IN VITRO* ANTIFUNGAL ACTIVITY

Time (h)	Tablet Conc. (μg/ml)	Gel Conc. (μg/ml)
1	18.78±1.038	21.15±0.8971
2	20.77±0.6322	21.06±0.9135
3	22.34±0.8447	21.15±0.8614
4	23.98±0.6373	
5	25.72±0.9030	
